# Spatialization of time in bilinguals: what do we make of the effect of the testing language?

**DOI:** 10.3389/fpsyg.2024.1355065

**Published:** 2024-06-12

**Authors:** Shuxia Cheng, Zhaohong Wu

**Affiliations:** ^1^School of English and International Studies, Beijing Foreign Studies University, Beijing, China; ^2^Division of Psychology and Language Sciences, University College London, London, United Kingdom

**Keywords:** English, Mandarin, mental representation of time, spatiotemporal conceptualization, linguistic context, the testing language, generalizability, bilingual

## Introduction

The question of how time is conceptualized spatially in human mind has received much academic interest over the years. Long-term linguistic and cultural effects on spatio-temporal conceptualization are found in many previous studies (e.g., Tversky et al., 1991; Fuhrman and Boroditsky, 2010; Boroditsky et al., 2011; Fuhrman et al., 2011; Miles et al., 2011; De la Fuente et al., 2014; Vallesi et al., 2014; Yang and Sun, 2016a,b; Li et al., 2018; Gu et al., 2019; Starr and Srinivasan, 2021; Grasso et al., 2022; Yang et al., 2022; but cf. Ulrich and Maienborn, 2010; Flumini and Santiago, 2013). For example, differences among languages in the use of spatiotemporal metaphors have been found to contribute to differences in spatialization of time in native speakers of different languages (e.g., Boroditsky, 2000, 2001; Boroditsky and Ramscar, 2002; Lai and Boroditsky, 2013; Núñez and Cooperrider, 2013; Bender and Beller, 2014). The more common use of vertical spatiotemporal metaphors in Mandarin than in English has been found to contribute to a significant difference between English and Mandarin speakers in their spatialization of time, such that Mandarin speakers are more likely than English speakers to think about time vertically, suggesting the power of long-term linguistic experience in influencing spatialization of time (e.g., Boroditsky et al., 2011; Fuhrman et al., 2011). Similarly, differences among languages in writing direction has been shown to affect the spatiotemporal conceptualization of time such that people's space-time mapping tends to be consistent with the writing direction of their native language (e.g., Tversky et al., 1991; Fuhrman and Boroditsky, 2010; Chen et al., 2013; Casasanto and Bottini, 2014; Vallesi et al., 2014; Starr and Srinivasan, 2021; Park et al., 2024). For example, spatialization of time on the transverse/lateral (left-right) axis is left-to-right for native speakers of English with a left-to-right writing direction, but right-to-left for native speakers of Arabic with a right-to-left writing direction (e.g., Tversky et al., 1991).

A growing line of research focus on how bilinguals conceptualize time spatially, in order to examine whether the spatiotemporal conceptualization of time in bilinguals may be influenced by the linguistic and cultural background of the L1 and/or the L2 (e.g., Boroditsky, 2001; Fuhrman et al., 2011; Miles et al., 2011; Lai and Boroditsky, 2013; Yang et al., 2022; Park et al., 2024), with the study population of interest being overwhelmingly Mandarin-English bilinguals. Previous research has revealed that the proficiency of Mandarin significantly affects spatiotemporal conceptualization of Mandarin-English bilinguals such that higher proficiency in Mandarin is associated with a higher probability of the vertical (top/down) representation of time (Fuhrman et al., 2011). In contrast, the proficiency of English does not significantly affect spatialization of time in Mandarin-English bilinguals (e.g., Fuhrman et al., 2011; Yang et al., 2022). In other words, it seems that achieving higher-proficiency in L2 English does not restructure Mandarin-English bilinguals' spatialization of time, neither strengthening the sagittal mental timeline nor weakening the vertical mental timeline (e.g., Yang et al., 2022).

Limited research has investigated whether immediate linguistic or cultural experience influence bilinguals' time conceptualization, by manipulating the testing language (e.g., Fuhrman et al., 2011, Experiment 2; Park et al., 2024) or the writing direction of the same language that participants are exposed to in actual experiments (e.g., Chen et al., 2013; Casasanto and Bottini, 2014) or the cultural-specificity of experimental items (e.g., Miles et al., 2011). Among those studies, Fuhrman et al.'s (2011) Experiment 2 and Park et al. (2024) are similar in demonstrating an effect of the testing language on spatialization of time in bilingual speakers, but different in the nature of the effect. In this article we examine the nature of the effects of the testing language in Fuhrman et al.'s (2011) Experiment 2 and Park et al. (2024) and evaluate the generalizability and implication of their findings for the literature on spatialization of time in bilinguals.

### Differences in the nature of the effects of the testing language in Fuhrman et al.'s (2011) Experiment 2 vs. Park et al. (2024)

In Fuhrman et al.'s (2011) Experiment 2, Mandarin-English bilinguals participated in an explicit spatial pointing task. In this task, the experimenter's gesture of the palm up and the fingers together, about a foot directly in front of the chest of the participant, was used to select a spot in space that corresponded to a central temporal event (e.g., *today, lunch, September*, etc.). Participants were then asked to spatially point to where they would put an earlier (e.g., *yesterday* with respect to *today, breakfast* with respect to *lunch, August* with respect to *September*, etc.) or later (e.g., *tomorrow* with respect to *today, dinner* with respect to *lunch, October* with respect to *September*, etc.) temporal event. Materials in this task were all culturally-neutral. Their results showed that Mandarin-English bilinguals were more likely to perceive time vertically when the testing language was Mandarin than when the testing language was English. Such an effect was linguistic in nature as native Mandarin speakers tend to represent time vertically more than native English speakers due to the more frequent use of vertical spatiotemporal metaphors in Mandarin (e.g., Boroditsky et al., 2011; Fuhrman et al., 2011). Fuhrman et al.'s (2011) finding of the effect of the testing language is then due to either Mandarin-English bilinguals' vertical mental timeline being strengthened with Mandarin as the testing language, or its being weakened with English as the testing language. We consider the former probability to be more likely as the more long-term effect of L2 English proficiency has been shown not to cognitively restructure Mandarin-English bilinguals' spatiotemporal conceptualization (Yang et al., 2022), so the immediate effect of English being the testing language seems even more unlikely to influence the spatialization of time in Mandarin-English bilinguals. In other words, it appears that Mandarin as the testing language help remind Mandarin-English bilinguals, either consciously or subconsciously, of the abundant vertical spatiotemporal metaphors in Mandarin and the consequent higher tendency to represent time vertically. In addition, Fuhrman et al.'s (2011) Experiment 2 also observed a significant cultural effect of experience with vertical text such that participants were less likely to arrange time in the lateral rightward direction with at least some experience of reading vertical texts.

Park et al. (2024) examined instead Arabic-English bilinguals' spatialization of time via a card arrangement task in which participants spatially arranged different scenes of a story on a table according to temporal sequence. They found an effect of the testing language for Arabic-English bilinguals' mental representation of time on the lateral axis such that they were more likely to arrange time from right to left when they were tested in Arabic than when they were tested in English. Such an effect was not linguistic but instead cultural, as it was not an effect of the Arabic language *per se*, but the writing direction of Arabic, which was a cultural artifact. In other words, being tested in Arabic seems to remind Arabic-English bilinguals of the right-to-left direction of writing associated with the Arabic language, either consciously or subconsciously. The effect of the testing language in Park et al. (2024) is due to either Arabic-English bilinguals' lateral right-to-left mental timeline being strengthened with Arabic as the testing language, or Arabic-English bilinguals' lateral right-to-left mental timeline being weakened with English as the testing language. Park et al.'s (2024) results were thus similar to previous studies that manipulated the immediate writing direction of texts (e.g., Chen et al., 2013; Casasanto and Bottini, 2014) but different in that Park et al.'s (2024) finding was an indirect mediating effect of long-term writing direction experience conjured up by the testing language, whereas the effects of writing direction in previous studies were direct effects of writing direction from either long-term or immediate reading experience (e.g., Fuhrman and Boroditsky, 2010; Vallesi et al., 2014).

### The issues of generalizability considering the effect of the testing language

It is important to note that the different tasks that have been employed in the literature to examine participants' spatiotemporal representation vary in their degree of implicitness (Bender and Beller, 2014). Both the spatial pointing task in Fuhrman et al.'s (2011) Experiment 2 and the card arrangement task used in Park et al. (2024) explicitly ask participants to spatially arrange time events, so participants' spatiotemporal representation as revealed by their spatial pointing or layouts of the time events is theoretically confounded with the task's inherent spatial nature (Bender and Beller, 2014), and participants' responses could be contaminated by their potential awareness of the true purpose of the study and/or possible testing strategies. In contrast, non-linguistic implicit temporal judgment tasks based on congruency priming (e.g., Fuhrman and Boroditsky, 2010, Experiment 2 and 3; Chen et al., 2013; Vallesi et al., 2014) examine participants' implicit space-time mapping through the spatial-temporal association between response codes (the “STARC” effect). How participants conceptualize time spatially is measured indirectly by analyzing their response time to different mappings either congruent or incongruent with their inner spatiotemporal conceptualization. Data elicited from such tasks are less likely to be affected by subjective testing strategies of the participants, and such tasks are less likely to make participants aware of the true purpose of the study. Moreover, as implicit contingency tasks are not spatial in nature, they could yield “the true extent of space-time mapping” (Bender and Beller, 2014, p. 365).

The influence from long-term linguistic experience with vertical spatiotemporal metaphors has been shown to apply to not only explicit tasks but also the implicit contingency tasks such that Mandarin speakers but not English speakers showed a vertical top-to-bottom STARC effect, consistent with the higher frequency of vertical spatiotemporal metaphors in Mandarin than in English (e.g., Fuhrman et al., 2011, Experiment 1). Similarly, the influence from long-term experience with the writing direction of the native language has been shown to apply to the STARC task for native speakers. For example, participants from a left-to-right writing system displayed a left-to-right STARC effect (e.g., English speakers in Fuhrman and Boroditsky, 2010; Italian speakers in Vallesi et al., 2014), whereas participants mainly from a right-to-left writing system did not demonstrate the STARC effect or a right-to-left STARC effect (e.g., Hebrew speakers in Fuhrman and Boroditsky, 2010; Israeli speakers in Vallesi et al., 2014).

It is therefore important to examine whether the immediate effect of the testing language would also affect time spatialization in bilinguals in implicit contingency tasks. To the best of our knowledge, no research so far has investigated such an issue. Previous evidence from Chen et al. (2013) on Chinese participants from both mainland China and Taiwan, however, suggest the possibility of such an immediate effect of the testing language. Participants from mainland China and Taiwan differ in their lifetime experience with writing direction, as vertical texts are fairly common in Taiwan but not in mainland China (Chen et al., 2013). Chen et al. (2013) manipulated participants' immediate experience with writing direction by asking participants to read Chinese texts that were either horizontally or vertically arranged in a reading task. In the subsequent STARC task, participants were asked to judge with horizontally- or vertically-oriented keys whether the event depicted in the second picture happened earlier or later than the event depicted in the first picture. Chen et al. (2013) revealed effects of lifetime experience with writing direction such that Taiwan participants were shown to have a greater vertical bias as compared to the horizontal bias, but mainland China participants showed no difference in the horizontal and vertical mental timelines. More importantly, Chen et al. (2013) also found an interaction between immediate and lifetime experiences with writing direction in modulating participants' mental timelines, arising from a stronger vertical bias after vertical primes but no vertical bias after horizontal primes for Taiwan participants, and a stronger horizontal bias after vertical primes but no horizontal or vertical bias after horizontal primes for mainland China participants. The significant immediate effects of writing direction found in Chen et al. (2013) suggest that the immediate effect of the testing language found with an explicit task for bilingual speakers of two languages with opposing writing direction, i.e., Arabic-English bilinguals in Park et al. (2024), may apply to the STARC task as well. It remains as well a standing issue whether the immediate linguistic effect of the testing language *per se* due to differences between languages in their use of spatiotemporal metaphors, as in Fuhrman et al.'s (2011) Experiment 2, would likewise arise in STARC tasks for bilinguals.

The significant interaction between long-term and immediate effects of writing direction in Chen et al. (2013) suggests a potential complication of the testing language on the long-term linguistic effects. As most previous studies on long-term linguistic effects did not control for the factor of the testing language, findings regarding long-term linguistic effects may have been confounded with the potential effect of immediate linguistic experience, which awaits future investigations.

## Conclusion

Only two studies (Fuhrman et al., 2011, Experiment 2; Park et al., 2024) so far have examined the immediate effect of the testing language on bilinguals' spatiotemporal conceptualization. In this article we have examined the nature of this effect in those two studies and pointed out that both studies employed explicit spatial tasks, leaving open the question whether this same effect would be generalizable to implicit contingency tasks as well.

## Author contributions

SC: Resources, Formal analysis, Writing – original draft, Investigation, Conceptualization. ZW: Resources, Writing – review & editing, Supervision, Formal analysis.
